# Unveiling the Fatigue Behavior of 2D Hybrid Organic–Inorganic Perovskites: Insights for Long‐Term Durability

**DOI:** 10.1002/advs.202303133

**Published:** 2023-07-06

**Authors:** Doyun Kim, Eugenia S. Vasileiadou, Ioannis Spanopoulos, Xuguang Wang, Jinhui Yan, Mercouri G. Kanatzidis, Qing Tu

**Affiliations:** ^1^ Department of Materials Science & Engineering Texas A&M University College Station TX 77840 USA; ^2^ Department of Chemistry Northwestern University Evanston IL 60201 USA; ^3^ Department of Chemistry University of South Florida Tampa FL 33620 USA; ^4^ Department of Civil & Environmental Engineering University of Illinois Urbana‐Champaign Urbana IL 61801‐2352 USA

**Keywords:** 2D hybrid organic–inorganic perovskites, failure behaviors, fatigue, in‐plane, static dwelling

## Abstract

2D hybrid organic–inorganic perovskites (HOIPs) are commonly found under subcritical cyclic stresses and suffer from fatigue issues during device operation. However, their fatigue properties remain unknown. Here, the fatigue behavior of (C_4_H_9_‐NH_3_)_2_(CH_3_NH_3_)_2_Pb_3_I_10_, the archetype 2D HOIP, is systematically investigated by atomic force microscopy (AFM). It is found that 2D HOIPs are much more fatigue resilient than polymers and can survive over 1 billion cycles. 2D HOIPs tend to exhibit brittle failure at high mean stress levels, but behave as ductile materials at low mean stress levels. These results suggest the presence of a plastic deformation mechanism in these ionic 2D HOIPs at low mean stress levels, which may contribute to the long fatigue lifetime, but is inhibited at higher mean stresses. The stiffness and strength of 2D HOIPs are gradually weakened under subcritical loading, potentially as a result of stress‐induced defect nucleation and accumulation. The cyclic loading component can further accelerate this process. The fatigue lifetime of 2D HOIPs can be extended by reducing the mean stress, stress amplitude, or increasing the thickness. These results can provide indispensable insights into designing and engineering 2D HOIPs and other hybrid organic–inorganic materials for long‐term mechanical durability.

## Graphical abstract

Few‐layer 2D hybrid organic–inorganic perovskites manifest better fatigue resilience than polymers under cyclic stresses. They tend to behave like ductile materials at low stress levels, but brittlely at high stresses, probably owing to an unexpected plastic deformation. The fatigue lifetime can be extended by reducing the mean stress, stress amplitude, or increasing the thickness.

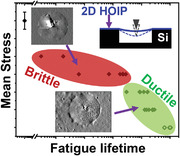

## 1 Introduction

The past decade has witnessed the rapid rise of hybrid organic–inorganic perovskites (HOIPs) as low‐cost, high‐performance semiconductor materials^[^ ^1^, ^2^ ^]^ with tremendous application potential across numerous semiconductor fields, including photovoltaics,^[^ ^3^, ^4^ ^]^ light‐emitting diodes,^[^ ^5^ ^]^ photo/radiation detectors,^[^ ^6^, ^7^ ^]^ and transistors.^[^ ^8^ ^]^ The relative softness of the lattice owing to the hybrid organic–inorganic nature,^[^ ^9^ ^]^ along with the great semiconductor performance, allows HOIPs to be incorporated into flexible and wearable electronics to greatly promote the advances in the Internet of Things.^[^ ^10^, ^11^, ^12^ ^]^ 2D HOIPs can be structurally derived from their 3D counterparts,^[^ ^2^, ^13^ ^]^ which further improve the materials’ chemical stability^[^ ^13^ ^]^ and mechanical resilience,^[^ ^9^, ^14^, ^15^, ^16^, ^17^ ^]^ and offer more tunability in the structure and chemistry for material properties engineering to meet various device application needs.^[^ ^9^, ^13^ ^]^

Mechanical strain is universally found in HOIP‐based devices.^[^ ^9^ ^]^ Owing to their soft nature, HOIPs are highly susceptible to influences arising from the strain, such as electronic band structure changes,^[^ ^18^ ^]^ ion migration,^[^ ^19^ ^]^ phase transition,^[^ ^20^ ^]^ and cohesive/adhesive failure.^[^ ^21^, ^22^ ^]^ Hence, it is crucial to understand the mechanical behavior of these materials to mitigate the unwanted strain effects^[^ ^9^ ^]^ and/or to strain engineer HOIPs to achieve better functionality or stability.^[^ ^20^, ^23^, ^24^ ^]^ As a result, the structure‐property relationship of HOIPs (both in 3D and 2D forms) regarding their elastic and fracture properties have been widely explored over the past few years,^[^ ^9^ ^]^ where the materials are typically loaded under quasi‐static conditions and simultaneous mechanical responses are recorded. However, in practical applications, HOIPs are more commonly found under subcritical (below the fracture strength, e.g., during strain engineering)^[^ ^23^, ^24^ ^]^ and cyclic loadings (e.g., during repeated bending in flexible electronics applications^[^ ^10^, ^11^, ^12^ ^]^ or thermal strain arising from temperature fluctuations).^[^ ^21^ ^]^ Materials typically suffer from mechanical fatigue under such loading conditions^[^ ^25^ ^]^ over the long service life of the device (ideally 10 to 20 years),^[^ ^26^ ^]^ and it is imperative to understand this behavior to evaluate the long‐term mechanical reliability. Accurately predicting the lifetime of components is crucial to replace them before catastrophic failure occurs. However, the lack of critical fatigue information for HOIPs impedes the design of mechanically robust HOIP‐based devices.

Due to the common presence of cyclic strain in device and composite applications,^[^ ^27^, ^28^, ^29^ ^]^ the fatigue properties of many low dimensional materials have been reported, including graphene,^[^ ^30^ ^]^ transition metal dichalcogenides,^[^ ^31^ ^]^ carbon nanotubes,^[^ ^32^, ^33^, ^34^ ^]^ Si nanowires,^[^ ^35^ ^]^ and SrTiO_3_ thin films.^[^ ^36^ ^]^ Despite the significant advancements in understanding the fatigue behavior of inorganic materials, the fatigue properties of hybrid organic–inorganic materials with reduced dimensions have received limited attention. Due to their hybrid nature, these materials can exhibit mechanical behaviors that are substantially different from both pure inorganic and pure organic materials, as demonstrated in their quasi‐static behaviors.^[^ ^9^, ^16^ ^]^ It would be fundamentally intriguing to see if, how and why 2D HOIP, a representative example of hybrid materials, will be different from other inorganic low dimensional materials under fatigue loading.

Here, we systematically investigate the fatigue behavior of 2D HOIPs membranes dynamically stretched by atomic force microscopy (AFM). We focus on a prototypical Ruddlesden–Popper 2D HOIPs, (C_4_H_9_‐NH_3_)_2_(CH_3_NH_3_)_2_Pb_3_I_10_ (abbreviated as C4n3 below), as a model example, which has been widely used for device demonstrations.^[^ ^2^, ^13^, ^37^ ^]^ The quasi‐static mechanical behavior of C4n3 has been well understood,^[^ ^14^, ^38^ ^]^ which can be compared to the dynamic mechanical behavior studied here. We discover that 2D HOIPs can exhibit better fatigue resistance than polymeric materials and the fatigue lifetime can reach 1 billion cycles. An unexpected plastic deformation mechanism in this material is revealed, which is likely responsible for this fatigue resilience. We further measure the quasi‐static mechanical properties after cyclic loading and uncover a progressive damage behavior. We find that cyclic loading can significantly accelerate the defect generation and accumulation in 2D HOIPs compared to static dwelling. We also unravel how the static and cyclic components of the loading, as well as the geometric factor (thickness) affect the fatigue lifetime of the membrane. Our findings offer invaluable insights into the fatigue properties of HOIPs and can aid in designing HOIP‐based devices with long‐term mechanical durability. Furthermore, as the structure‐property relationship of other hybrid organic–inorganic materials, such as metal–organic framework materials, may share similarities, our study may provide valuable knowledge regarding the fatigue behavior of these materials as well.

## 2 Results and Discussion

### 2.1 Fatigue Test of Thin 2D HOIP Membranes

**Figure 1:**
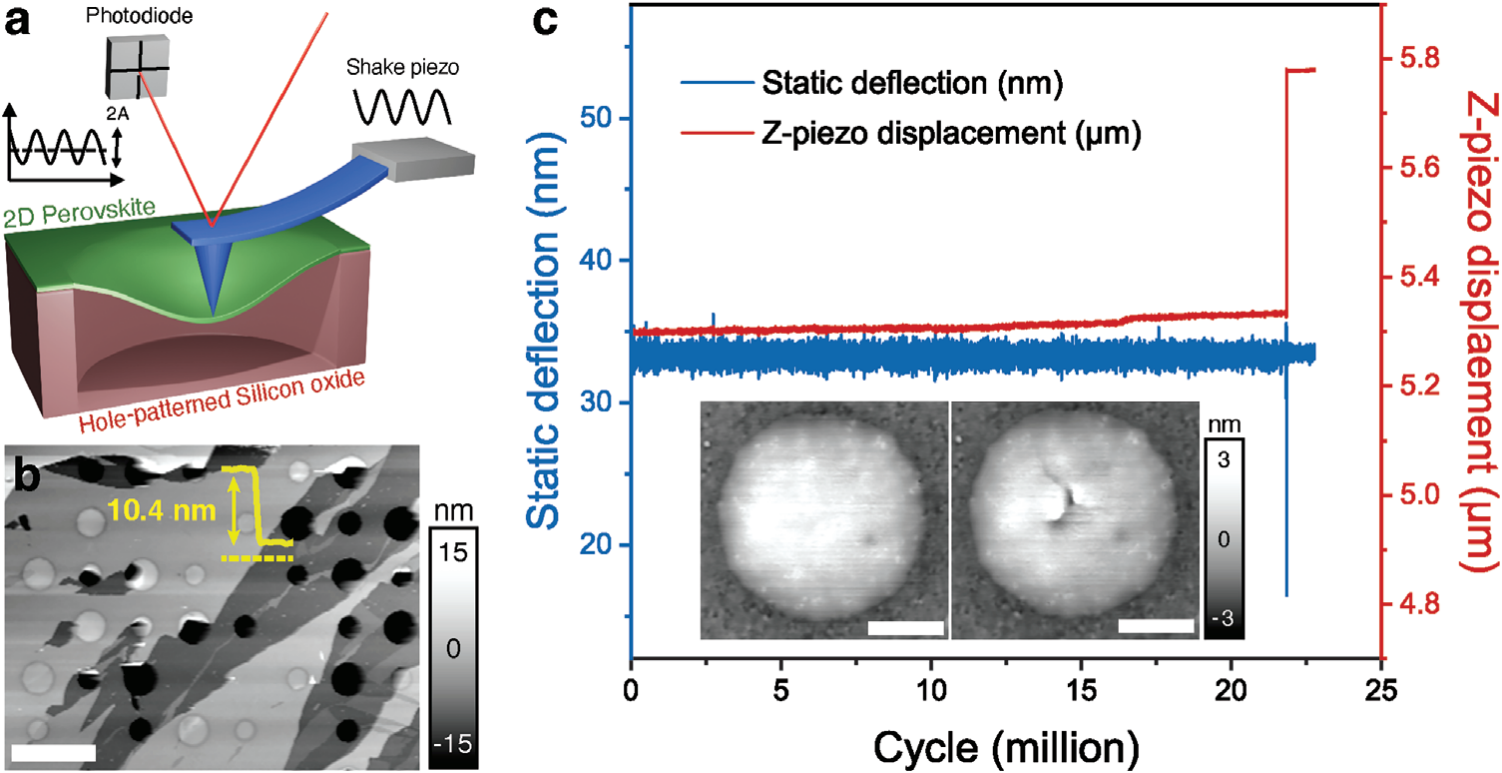
AFM‐based fatigue test of 2D HOIPs: a) schematic of AFM‐based fatigue method used in this study. b) Four‐layer thin C4n3 membrane deposited on hole‐patterned silicon oxide substrate. Inset: the measured height profile along the yellow dashed line showing the thickness of the flake. Scale bar: 4 µm. c) Representative fatigue data showing static deflection and displacement change as a function of cycles, where the fatigue failure of the membrane is indicated by the sharp changes in recorded cantilever deflection and *z*‐piezo displacement. Inset: AFM topographic images of the C4n3 membrane showing before (left) and after (right) fatigue failure. Scale bar: 400 nm.

AFM‐based fatigue test is employed here to investigate the fatigue behavior of thin 2D HOIP membranes (**Figure** **1**a). The method slightly modifies the quasi‐static in‐plane stretching test of 2D HOIPs membranes that was established in our group.^[^ ^14^, ^17^, ^38^ ^]^ Briefly, 2D HOIP C4n3 (Figure S1, Supporting Information (SI) for the structure schematic) thin membranes are mechanically exfoliated from solution‐grown single crystals and transferred to hole‐patterned silicon wafer using scotch tapes (see Experimental Section and SI ‐ Section I for more details). The thin flakes are first identified by optical microscopy and then imaged by AFM in tapping mode (Figure 1b). The tapping mode images are used to measure the thickness of the membranes and to position the AFM tip to the center of the membrane for mechanical tests. For the fatigue test (Figure 1a, see Figure S3, SI ‐ Section II for details), the AFM tip oscillates around a mean force with a force amplitude. The mean force is controlled by the static deflection of the AFM cantilever, while the oscillation force amplitude is controlled by the shake piezo and monitored by a lock‐in amplifier. We term the static and cyclic components of the forces as *F*_dc_ and *F*_ac_, respectively, as an analogue to the direct/alternating current of electricity. Apparently, *F*_dc_ and *F*_ac_ will provide controls over the mean stress and stress amplitude applied to the membrane during the fatigue test. The maximum force during each cycle, i.e., *F*_dc_ + *F*_ac_, are kept below the fracture force of the membrane. When the membranes fail under the dynamic loading, a sudden jump in the DC deflection, *z*‐piezo, and the AC amplitude will appear (Figure 1c and Figure S4 in SI) and the total number of fatigue cycles the membrane survives under the testing conditions can be derived. Similar AFM dynamic mechanical tests have been successfully utilized to uncover the fatigue behavior of other 2D materials and nanowires.^[^ ^30^, ^31^, ^35^, ^36^, ^39^, ^40^, ^41^ ^]^

To avoid the influences stemming from any frequency‐dependent behavior of the materials (e.g., ion migration) on the tested results and the comparisons afterward, we fix the dynamic loading frequency to 100 kHz for all fatigue tests here. This frequency is two orders of magnitude or more below the first fundamental resonance frequency of the C4n3 membranes (See Section II.2 in SI for detailed analysis), and the AFM tip oscillation amplitude (≤ 3 nm) is also much lower than the DC loading‐induced deformation of the membranes.^[^ ^14^ ^]^ Hence, the tip should continuously contact the 2D HOIP membranes during the oscillation.^[^ ^30^ ^]^ Here we also focus on the fatigue behavior of 2D HOIPs ≥ 3 layers, whose mechanical properties can represent that of bulk materials where the effects of sliding between the 2D layers at the van der Waals (vdW) interfaces on the mechanical behavior will be saturated.^[^ ^14^ ^]^ 2D HOIPs are widely reported to be much more stable than their 3D analogs owing to the inclusion of hydrophobic organic spacer molecules in the crystal structure.^[^ ^9^, ^13^, ^42^ ^]^ We have previously demonstrated that molecularly thin 2D HOIPs are stable for 12 h under relative humidity (RH) of 20%–30%,^[^ ^14^ ^]^ which should last much longer as RH further goes down.^[^ ^42^ ^]^ To ensure the membrane quality throughout the experimental window and to avoid potential damage of the membranes caused by the high humidity in our lab ambient environment (≈45%), the sample preparation (e.g., exfoliation and transfer of C4n3 membranes) are performed in a dry box (RH < 5%) and the AFM measurements are conducted in a controlled environment under dry air flow (RH < 3%). This is particularly important for the fatigue experiments at low *F*_dc_ due to the extended experimental time.

### 2.2 Fatigue Behavior under Cyclic Loading

**Figure 2:**
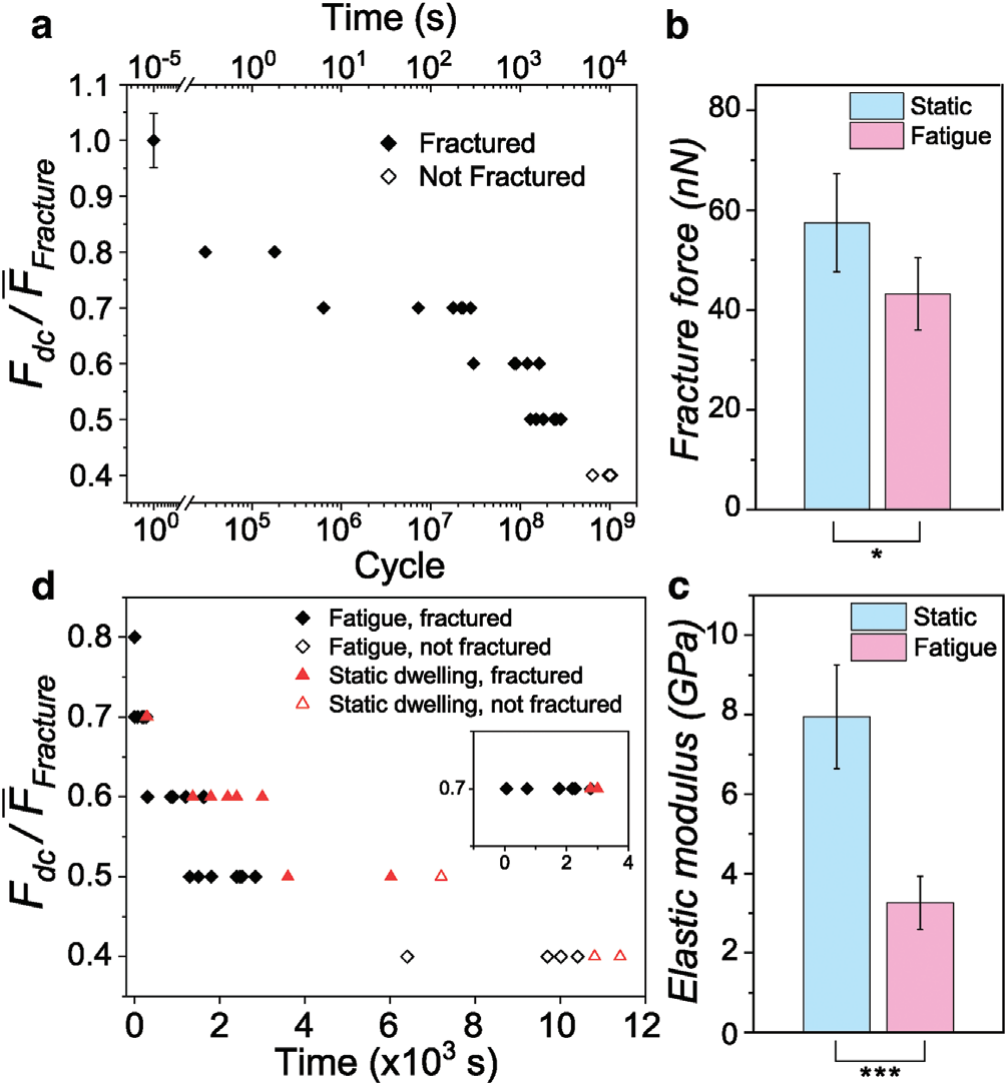
Fatigue and static‐dwelling of 4‐layer C4n3 2D HOIP membranes: a) number of fatigue cycles survived under various $F_{\mathrm{dc}}/{\bar{F}}_{\mathrm{fracture}}$ (≈ 1 µm diameter, 0.75 nm tip amplitude) showing increasing lifetime at lower average force. Sample sizes are 23, 2, 6, 6, 7, and 5 for *F*_dc_ = 100%, 80%, 70%, 60%, 50%, and 40% of ${\bar{F}}_{\mathrm{fracture}}$, respectively. b,c) Are the fracture force and elastic modulus, respectively, of the membranes after 3 million cycles cyclic loaded at $F_{\mathrm{dc}}=\;60\%{\bar{F}}_{\mathrm{fracture}}$ compared to those from the pristine membranes. Sample sizes are 6 and 5 for (b) and (c), respectively. “*” and “***” indicate *P* ≤ 0.05 and < 0.001, respectively. d) Lifetime of the membranes under static (red) and cyclic (black) loading under various $F_{\mathrm{dc}}/{\bar{F}}_{\mathrm{fracture}}$. Inset: Fatigue and static dwelling data of $70\%{\bar{F}}_{\mathrm{fracture}}$ showing longer lifetime under static dwell.

We first monitor the number of fatigue life cycles that 4‐layer (thickness ≈ 10.4 ± 0.1 nm,^[^ ^14^ ^]^ Figure 1b) of C4n3 membranes can survive under a constant *F*_ac_ as we vary *F*_dc_ (**Figure** **2**a). To facilitate the comparison of the results from different samples, we have normalized *F*_dc_ by the average fracture forces ${\bar{F}}_{\mathrm{fracture}}$ of the 2D HOIP membranes, similar to earlier studies of the fatigue behavior of other 2D materials.^[^ ^30^, ^31^, ^40^, ^41^ ^]^ The oscillation amplitude of the AFM tip is first fixed to 750 pm, which corresponds to ≈1.5 nN force amplitude, much lower than even the lowest *F*_dc_ we apply during the test (≈23 nN) to ensure the continuous contact of the tip‐sample during the oscillation. The estimated strain amplitude of the oscillation is ≈0.1% (see Section II.3 in SI). The strain amplitude is equivalent to thermal strains induced by ≈10 to 20 °C temperature fluctuations, which 2D HOIPs can commonly encounter during device operation^[^ ^21^, ^26^, ^43^ ^]^ (Section II.3 in SI for detailed analysis). The strain amplitude also falls in the range of strains that the materials will experience during flexible electronics applications (Section II.3 in SI). Hence, the results reported here are quite technologically relevant and can provide indispensable guidance to mitigate mechanical failure issues for 2D HOIP‐based device applications.

When we load the membrane to its fracture force, it will immediately fail, resulting in zero cycles survival in the cyclic loading period (Figure 2a). At $F_{\mathrm{dc}}=\;80\%\;{\bar{F}}_{\mathrm{fracture}}$, 4‐layer C4n3 membranes can survive up to 1.79 × 10^5^ cycles. As we decrease *F*_dc_ to $70\%\;{\bar{F}}_{\mathrm{fracture}}$, the membranes can survive (1.63 ± 0.84) × 10^7^cycles (at 95% confidence), which is beyond the need of most practical engineering applications (> 10^6^ cycles).^[^ ^44^ ^]^ Further lowering *F*_dc_ results in a rapid increase in the fatigue lifetime of the membrane (Figure 2a). For instance, at $F_{\mathrm{dc}}=\;60\%\;{\bar{F}}_{\mathrm{fracture}}$ and $50\%\;{\bar{F}}_{\mathrm{fracture}}$, the membrane can survive (1.08 ± 0.41) × 10^8^ and (2.12 ± 0.44) × 10^8^ cycles, respectively. These results clearly demonstrate the strong mean stress effect on the fatigue lifetime of 2D HOIPs, which is consistent with the fatigue behavior of classical bulk materials.^[^ ^25^ ^]^ More interestingly, when the mean force is at $40\%\;{\bar{F}}_{\mathrm{fracture}}$, no fatigue failure is observed even after 1 billion cycles, where the estimated mean stress and stress amplitude is ≈220 and 11 MPa, respectively (Section II.3 in SI). The measured fatigue lifetime of C4n3 2D HOIPs is much longer than many plastics under similar or more mild loading conditions, including polystyrene, polyethylene, polypropylene, Nylon, polyethylene terephthalate, polymethyl methacrylate, *etc*.^[^ ^45^, ^46^ ^]^ This demonstrates the extraordinary fatigue resistance of pristine C4n3 2D HOIP membranes and further endorses the great potential of 2D HOIP in flexible electronics applications.

To uncover the effect of cyclic loading on the mechanical properties of 2D HOIPs and further gain insights into the fatigue failure mechanism, we measure the elastic moduli and fracture force of the membranes after 3 million cycles under $60\%\;{\bar{F}}_{\mathrm{fracture}}$ following the method we developed before^[^ ^14^, ^17^, ^38^ ^]^ (see Section II.4 in SI ), and compare to the results obtained from the as‐prepared C4n3 membranes with the same thickness. For 4‐layer thick pristine 2D C4n3 membranes, the measured fracture force *F*_fracture_ and elastic modulus *E* are 57.4 ± 9.8 nN and 7.94 ± 1.30 GPa, respectively (Figure 2b,c). *E* is close to what we measured under similar experimental conditions in a separate report,^[^ ^38^ ^]^ showing the high quality and reproducibility of the pristine C4n3 membranes. After being cyclic loaded, the membranes become much weaker and softer. *F*_fracture_ and *E* drop by 25% and 58%, respectively (Figure 2b,c), which is consistent with the detrimental effect of cyclic bending on the fracture resistance found in polycrystalline HOIP thin films.^[^ ^47^ ^]^ However, this is in stark contrast to the insensitivity of the elastic and fracture properties found in brittle single‐crystalline materials like graphene^[^ ^30^ ^]^ and SrTiO_3_,^[^ ^36^ ^]^ but closer to the behavior of ductile materials, such as viscous plastic Al_2_O_3_ grown by atomic layer deposition (ALD)^[^ ^41^ ^]^ and graphene oxide (GO) with epoxide groups,^[^ ^30^, ^40^ ^]^ suggesting the presence of some plastic deformation mechanisms in 2D HOIPs.

The negative effect of cyclic loading on *E* is further confirmed by the decreasing trend of *E* as a function of survived cycles during the test (Figure S7 in SI). Therefore, cyclic stress significantly deteriorates the quasi‐static mechanical performance of 2D HOIPs, which eventually causes the material's failure and is probably due to the accumulation of defects induced by cyclic stress (e.g., nanovoids). Our results also demonstrate that 2D HOIPs exhibit cyclic softening behavior. Typically, ductile inorganic materials, e.g., metallic materials, show cyclic hardening and cyclic softening if they start at intrinsic state or strain hardened state, respectively.^[^ ^25^, ^46^ ^]^ In contrast, polymers only exhibit cyclic softening due to microstructure‐packing rearrangement and generation of certain types of defects induced by cyclic loading.^[^ ^46^ ^]^ Although the inorganic PbI_6_^4−^ framework can be strain hardened,^[^ ^48^ ^]^ the applied load in the incipient stage of the fatigue tests here is well below the elastic limit.^[^ ^14^ ^]^ On the other hand, owing to the hybrid organic–inorganic nature, 2D HOIPs and their 3D counterparts do manifest mechanical behaviors that resemble those of polymers under quasi‐static loading.^[^ ^9^, ^16^ ^]^ Our results suggest that such organic‐like mechanical behaviors also exist in 2D HOIPs under cyclic loading conditions.

To further understand the stochastic failure behavior of 2D HOIPs, we employ a two‐parameter Weibull distribution to analyze the survival probability of the tested C4n3 membranes under fatigue loading for 100 million cycles and compare to the quasi‐static failure case (Figure S8in SI). Such Weibull distribution has been widely used to understand the survival or failure of materials under both statical and cyclic loading conditions.^[^ ^30^, ^41^, ^49^, ^50^, ^51^ ^]^ The probability of survival as a function of force is given as

(1) $$P\left(F\right)\;=\;\exp\left[-{\left(\frac{F/{\bar{F}}_{\mathrm{fracture}}}{\lambda }\right)}^{m}\right]$$

where *λ* is a characteristic scaling factor associated with the probability distribution and *m* is the Weibull modulus, which describes the breadth of the distribution. *m* of C4n3 membranes is found to be 11.5 under quasi‐static fracture. Such high *m* is comparable to those from single crystalline graphene (13.9 to 16)^[^ ^30^, ^49^ ^]^ and SrTiO_3_ membranes,^[^ ^36^ ^]^ but much higher than the *m* values of nanotubes and nanowires,^[^ ^52^, ^53^ ^]^ confirming the high quality of the C4n3 2D HOIP membranes. The *m* value for C4n3 membranes to survive 100 million cycles is 10.5, only slightly lower than *m* from the quasi‐static failure case (Figure S8 in SI). In contrast, graphene shows a dramatic drop in *m* for survival under cyclic loading compared to that under quasi‐static loading.^[^ ^30^ ^]^ This suggests that compared to graphene, 2D HOIPs are less sensitive to the pre‐existing defects (chemically formed during growth or sample transfer/handling) in the materials under cyclic loading. The Weibull distribution result is also consistent with the fact that our fatigue failure data in C4n3 membranes is much less scattered than those from graphene. In addition, the result implies that the defects generated by cyclic loading are different from the pre‐existing defects because the cyclic loading‐induced defects cause significant deterioration of the mechanical properties of 2D HOIPs (Figure 2b,c; Figure S7 in SI) and shorten their fatigue lifetime (Figure 2a). The Weibull statistical analysis further corroborates that under cyclic loading, 2D HOIPs behave quite differently from graphene and transition metal dichalcogenides.

Owing to the ionic bonding nature, 3D HOIPs are widely recognized as brittle materials with little plastic deformation found in experiments, regardless of single crystalline or polycrystalline forms.^[^ ^9^ ^]^ Although the inclusion of organic spacer molecules and the presence of weak vdW interfaces in the 2D structured HOIPs can potentially enable plastic deformation through sliding at vdW interfaces,^[^ ^14^ ^]^ to achieve prominent plasticity through such interface sliding at few‐layer thicknesses, it requires the individual constituent layer of 2D materials to be plastically deformable as well, as suggested by early studies of brittle graphene and ductile GO.^[^ ^54^, ^55^, ^56^, ^57^, ^58^, ^59^, ^60^ ^]^ Therefore, it is generally expected that 2D HOIPs should be similarly brittle considering the extreme resemblance between the PbI_6_^4−^ frameworks in 3D and 2D HOIPs^[^ ^13^, ^61^ ^]^ (Figure S1in SI) and the dominant effect of the PbI_6_^4−^ framework on the in‐plane mechanical properties of 2D HOIPs.^[^ ^14^, ^17^ ^]^ Brittle 2D materials like graphene usually display global catastrophic failure of the membrane with long cracks^[^ ^30^ ^]^ while 2D materials with ductile features, such as GO with epoxy functional groups^[^ ^30^, ^40^ ^]^ and viscous‐plastic Al_2_O_3,_^[^ ^41^ ^]^ exhibit contained damage upon fatigue failure under AFM testing.

**Figure 3:**
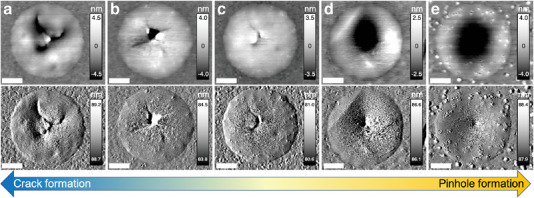
Morphology of the C4n3 membranes after fatigue failure at various *F*_dc_: a) 80%, b) 70%, c) 60%, d) 50%, and e) 40% of ${\bar{F}}_{\mathrm{fracture}}$. In (e), the membrane did not fail. Top and bottom rows are tapping mode AFM topographic and amplitude images, respectively. Scale bar: 300 nm.

Hence, we further examine the morphology of the C4n3 membranes after fatigue failure. Surprisingly, we notice both failure patterns in C4n3 membranes and the failure patterns depend on the mean force value (i.e., *F*_dc_) of the cyclic loading (**Figure** **3**). Under high *F*_dc_, the membranes tend to fail catastrophically like a brittle 2D material^[^ ^30^, ^31^ ^]^ (Figure 3a–c) while under low *F*_dc_, the damage tends to be contained locally with a small pinhole at the center of the membrane after failure (Figure 3d), which is more like the fatigue failure of ductile membranes.^[^ ^30^, ^40^, ^41^ ^]^ In fact, snapshots of the topography of the C4n3 membranes after various cycles of fatigue loading ( $F_{\mathrm{dc}}=\;50\%\;{\bar{F}}_{\mathrm{fracture}}$) before failure (Figure S9 in SI) clearly shows signs of progressive plastic damage similar to ALD Al_2_O_3_ thin films.^[^ ^41^ ^]^ For membranes loaded at $40\%\;{\bar{F}}_{\mathrm{fracture}}$, even though they maintain structural integrity after 1 billion cycles, plastic deformations are also found with a damage pin in the center and a large plastically deformed zone (Figure 3e). Our results suggest that a plastic deformation mechanism can be activated under low mean stress levels that could sufficiently dissipate the mechanical energy put into the system and arrest the crack propagation. However, such plastic deformation mechanism cannot contain the damage under large mean stress levels, indicating that high mean stresses probably deactivate or impede this plastic deformation mechanism. Numerous prior studies, as summarized in our recent review,^[^ ^9^ ^]^ have revealed that the stability of the optoelectronic properties and performance of HOIPs is adversely impacted by tensile strain, with the negative effect proportional to the magnitude of the strain. This finding aligns with the trend observed in our investigation of the fatigue behavior of 2D HOIPs. The detrimental effect of the mean stress level on the plastic deformation in 2D HOIPs is probably responsible for the dramatic drop in the fatigue lifetime as we increase the applied mean forces (Figure 2a). The fact that no signatures of vdW interfacial failure defects (e.g., wrinkles or delamination) in these 4‐layer C4n3 membranes (Figure 3; Figure S9 in SI) as found in few‐layer graphene^[^ ^30^ ^]^ implies that the plastic deformation mechanism might not mainly arise from the sliding at the vdW interface between the two organic spacer molecules, but rather from the deformation within each repeating unit (e.g., ion migration, self‐healing, or stress‐induced amorphization).^[^ ^9^ ^]^ The true nature of such plastic deformation mechanism and its dependence on the mean stress is currently under investigation in our group and is beyond the scope of this paper.

### 2.3 Failure under Static Dwelling

To further understand the cyclic loading effect, we also perform long‐term static dwelling of 4‐layer C4n3 membranes and compare the lifetime to the results under cyclic loading. Under this loading condition, no active oscillation is applied to the tip, but only thermal fluctuations cause an almost‐zero (average ≈ 15 pm, peak value ≈ 40 pm) tip amplitude (Figure S10 in SI). Our results in Figure 2d show that subcritical static dwelling can indeed cause structure failure in 2D HOIPs after a sufficiently long time, particularly at high *F*_dc_, which is similar to graphene.^[^ ^30^ ^]^ The duration required for sufficient damage accumulation leading to failure decreases as mean stresses increase, providing additional evidence of the harmful impact of tensile strain on the long‐term stability of these materials. However, unlike graphene where such failure is not observed <$76\%\;{\bar{F}}_{\mathrm{fracture}}$,^[^ ^30^ ^]^ C4n3 membranes continues to show static dwelling failure even at $50\%\;{\bar{F}}_{\mathrm{fracture}}$. No failure is found when the membranes are dwelled at $40\%\;{\bar{F}}_{\mathrm{fracture}}$ for a time more than the longest equivalent lifetime tested under cyclic loading conditions. Furthermore, the time needed to induce failure under cyclic loading is always shorter than that under static dwelling, which is quite dramatic considering the small amplitude of the oscillatory component (≈0.1% strain). Our results demonstrate that the kinetics that drives the structural failure under subcritical tensile stress can be accelerated by cyclic loading. However, the difference of the membranes’ lifetime under static dwelling versus cyclic loading shrinks as the mean force increases (Figure 2d). These results also suggest that static strain‐engineering of HOIPs for functional property/phase control should be kept at a low level to ensure long‐term stability of the materials.

**Figure 4:**
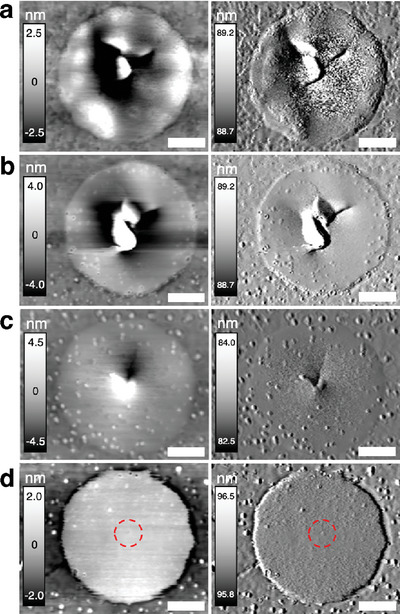
Morphology of the C4n3 membranes after static dwelling at various *F*_dc_: a) 70%, b) 60%, c) 50%, and d) 40% of ${\bar{F}}_{\mathrm{fracture}}$. In (d), the membrane maintains structural integrity and the red dashed circle marks the sign of damage at the center. Left and right columns are tapping mode AFM topographic and amplitude images, respectively. Scale bar: 300 nm.

We further characterize the morphology of the C4n3 membranes after static‐dwelling failure (**Figure** **4**). Similar to the cyclic loading case, high mean forces lead to brittle‐like failure (Figure 4a,b) while failure under low mean forces is ductile‐like (Figure 4c). Progressive damage during the static dwelling is also revealed by the topographic snapshots during the dwell test (Figure S11 in SI). The accumulated progressive damage is found in $40\%\;{\bar{F}}_{\mathrm{fracture}}$ (Figure 4d) statically dwelled membranes as well. However, the damage size and plastically deformed zone is much smaller than those found under cyclic loading (Figure 3e) at the same mean force, suggesting a much longer dwelling lifetime than the tested time here. The results also confirm the higher damage accumulation rate under cyclic loading than under static dwelling. Furthermore, we record *E* as a function of static dwelling time (Figure S7, Supporting Information) at $60\%{\bar{F}}_{\mathrm{fracture}}$ and compared to those from cyclic loading under the same mean force. Similar to cyclic loading, static dwelling also deteriorates *E*, but at a much lower rate, further confirming that cyclic loading can accelerate the damage nucleation and accumulation in 2D HOIPs.

### 2.4 Modulate the Fatigue Lifetime

**Figure 5:**
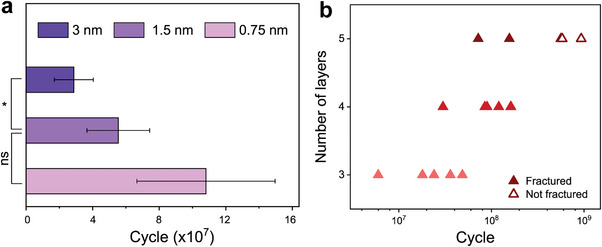
Fatigue lifetime of C4n3 2D HOIP membranes under a) different cyclic amplitude and b) at different thicknesses. In both cases, $F_{\mathrm{dc}}=\;60\%{\bar{F}}_{\mathrm{fracture}}$. Thickness in (a) is 4‐layer and the tip oscillation amplitude in (b) is 0.75 nm. The sample sizes for each thickness in (a) are 6. “ns” and “*” indicate not significant (*P* > 0.05) and *P* ≤ 0.05, respectively.

Besides the mean stress effect shown in Figure 2a, we further test the influence of the cyclic loading amplitude on the fatigue lifetime of 2D HOIPs by fixing *F*_dc_ to $60\%{\bar{F}}_{\mathrm{fracture}}$ and recording the fatigue lifetime of 4‐layer C4n3 as a function of *F*_ac_. When the tip oscillation amplitude is doubled (1.5 nm), the average fatigue lifetime drops from (10.8 ± 4.1) × 10^7^ to (5.54 ± 2.30) × 10^7^ (**Figure** **5**a). Further increasing the oscillation amplitude to 3 nm decreases the lifetime to (2.87 ± 1.40) × 10^7^. These results unambiguously confirm the negative effects of cyclic loading amplitude on the materials’ capability to resist fatigue failure. As discussed in the previous section, cyclic loading accelerates the damage accumulations in 2D HOIPs. Hence, larger cyclic loading amplitudes result in faster damage accumulations and thus shorter lifetime of 2D HOIP membranes. The observed trend of fatigue lifetime on the force amplitude is similar to those in other 2D materials. Our results suggest that besides the mean stress, the stress amplitude in 2D HOIPs should be kept low through device architecture design to avoid mechanical failures in the long term.

Lastly, we vary the thickness of the C4n3 membranes from 3‐ to 5‐layers (Figure S12 in SI) and measure their fatigue lifetime at $F_{\mathrm{dc}}=60\%{\bar{F}}_{\mathrm{fracture}}\;$ and 0.75 nm tip oscillation amplitude. Other 2D materials, such as graphene, GO, and graphene/Al_2_O_3_ composites, usually show shorter fatigue lifetime in thicker samples owing to higher defect densities in thicker samples (size effect). However, as shown in Figure 5b, thicker C4n3 membranes have longer fatigue lifetimes. Some 5‐layer C4n3 membranes can survive ≈1 billion cycles (Figure 5b) that are comparable to the lifetime of 4‐layer C4n3 membranes under *F*_dc_ = $40\%{\bar{F}}_{\mathrm{fracture}}$ and the same cyclic force amplitude (Figure 2a). The thickness‐dependent fatigue lifetime here suggests that the density of fatigue‐sensitive defects in these molecularly thin single crystals of C4n3 membranes does not significantly grow with the sample thickness, similar to the density of defects that can influence the quasi‐static mechanical properties of 2D HOIPs.^[^ ^14^ ^]^ While the defect density remains constant, thicker 2D HOIPs may facilitate more plastic deformations by incorporating additional layers capable of accommodating plastic deformation (as discussed earlier), thereby enhancing their resistance to fatigue failure.

## 3 Summary and Conclusion

In conclusion, we systematically studied the mechanical failure behavior of ultrathin 2D HOIP membranes under sub‐critical (i.e., below the strength) cyclic and static dwelling loading conditions. When $F_{\mathrm{dc}}=70\%{\bar{F}}_{\mathrm{fracture}}\;$, the membranes can survive over 1 million cycles, sufficient for engineering applications from the fatigue perspective, yet the absolute time they can last is still relatively short, owing to the high mean stress. The lifetime of the membranes quickly increases as the applied mean force drops in both cyclic and static dwelling cases, showing a strong mean stress effect on the fatigue and creep behaviors of these materials. At $40\%{\bar{F}}_{\mathrm{fracture}}$, the membranes can survive over 1 billion cycles, which outperforms most polymer materials. The applied subcritical tensile stress continuously deteriorates the elastic modulus and fracture strength of the materials, and the cyclic component of the loading can amplify the detrimental effects of tensile stress and significantly accelerate the deterioration process. The failure morphology of the membranes indicates that they tend to exhibit brittle failure at higher mean stress levels, whereas they behave as ductile materials at lower mean stress levels. These observations suggest the presence of a plastic deformation mechanism in this ionic hybrid organic–inorganic material at low mean stress levels, which may contribute to its extended lifetime, but is inhibited at higher mean stress levels. Moreover, the fatigue lifetime of the membranes reduces with increasing cyclic load amplitude, while thicker 2D membranes exhibit a longer fatigue lifetime, in contrast to other low‐dimensional materials. Our results suggest that 2D HOIPs can be mechanically robust under subcritical static and cyclic loadings in device applications, the device architecture must be designed such that the mean stress and cyclic stress will maintain at relatively low levels to ensure their long‐term durability. The unique fatigue behaviors found in 2D HOIPs here might also exist in other low‐dimensional materials with hybrid organic–inorganic nature, e.g., 2D metal organic frameworks, as suggested by the similarities of their mechanical behaviors,^[^ ^9^, ^38^, ^62^ ^]^ and call for further exploration into the structure‐fatigue property relationship of materials with hybrid features.

## 4 Experimental Section

### Materials

C4n3 2D HOIP single crystals are directly grown from solution following the protocols developed before^[^ ^15^, ^61^ ^]^ (details in Section SI.1 in SI). The synthesized 2D HOIP single crystals are characterized by powder X‐ray Diffractometer. The obtained diffraction patterns are compared to the calculated pattern from the reported single crystal structure (Section SI.2  in SI) to confirm the phase purity. Extensive analysis of the single crystal structures of the 2D HOIPs was reported elsewhere.^[^ ^61^ ^]^

### Preparation of Suspended 2D HOIP Membranes

The hole arrays on SiO_2_/Si were fabricated by photolithography as described in the earlier studies.^[^ ^14^, ^17^, ^38^ ^]^ Prior to the C4n3 thin membrane transfer, the hole‐patterned silicon wafer is first sonicated with ethanol, and further washed with freshly prepared piranha solution (3:1 by volume for 98% H_2_SO_4_ and 35% H_2_O_2_). The wafer is then rinsed thoroughly with deionized water and dried with compressed dry air. The C4n3 membranes are then directly exfoliated from the as‐grown single crystals to the hole‐patterned silicon wafer via the classical scotch tape method in a dry box (RH < 5%, filled with compressed dry air). The prepared silicon wafer with deposited membranes is transferred by a petri dish containing silica gel and secured under AFM head in AFM chamber filled with compressed dry air for AFM characterizations.

### AFM Fatigue and Static Dwelling Test

All AFM tests are conducted under constant dry air flow (RH < 3%) with an Asylum MFP‐3D Infinity AFM (Asylum Research, an Oxford Instrument) enclosed by a plastic bag around the AFM head and sample stage. It is observed no apparent changes in the surface morphology during experiments that lasts up to 15 h, indicating high stability of 2D HOIP membranes in the experimental environment. To minimize tip wear during the extended experiments, diamond‐like‐carbon coated tips (AIODLC cantilever B, BudgetSensor) are used for all AFM tests here. The tip radius change before and after the fatigue tests by SEM is closely monitored, where the tip radius shows negligible change (<10%) (Figure S13 in SI). Prior to the AFM measurements, the deflection sensitivity of the AFM cantilever is calibrated by engaging the cantilever onto a clean silicon wafer. The spring constant of the cantilever is calibrated by measuring the power spectral density of the thermal noise fluctuations in the air via fitting the first free resonant peak to the equations of a simple harmonic oscillator.^[^ ^63^, ^64^ ^]^

The average fracture force of C4n3 membranes for each thickness is measured by quasi‐statically loading the suspended membranes (from multiple exfoliated flakes) to fracture. These fracture tests  give us a good idea of the fracture force distribution for high‐quality samples. To avoid the influence of high‐density pre‐existing defects in the 2D HOIPs (e.g., due to aging of the surface layers of the 2D HOIP crystals or mishandling during the transfer), the exfoliated 2D HOIP membrane quality is pre‐screened by measuring the fracture forces of at least three membranes on the same large C4n3 flakes (Figure 1b; Figure S12 in SI) and comparing the results to the obtained fracture force distribution of C4n3 flakes with the same thickness before performing fatigue tests of a membrane on the same C4n3 flakes. The lateral drift level in the AFM system (typically a few hundred pm/min, as measured by the AFM) is higher than that reported in fatigue studies of graphene,^[^ ^30^ ^]^ despite the long stabilization period (3 to 5 h) before the experiments, owing to the requirement of continuous air flow for the 2D HOIP experiment. Hence, the fatigue and static dwell experiments are conducted in many short segments until the membrane fails or the total experiment time gets too long. Each segment lasts for a few minutes such that within this segment, the drift distance would be less than a quarter of the tip radius to avoid any drift‐induced damage to the membranes. After each segment, the membranes are reimaged and the tip isrepositioned to the membrane center to compensate the drift, which also allows it to keep track of the mechanical property change during the fatigue or the static dwelling experiments. This drift mitigation method was employed in AFM‐based fatigue studies of nanowires.^[^ ^35^ ^]^

### Statistics

Statistical data in all experiments is presented as mean ± 2× the standard error of the mean with all sample sizes mentioned in the corresponding figure captions. Microsoft Excel is used to perform the statistical analyses. Student *t* test is used to evaluate the statistical significance of different experimental conditions.

## Conflict of Interest

The authors declare no conflict of interest.

## Supporting information

Supporting Information

## Data Availability

The data that support the findings of this study are available from the corresponding author upon reasonable request.
