# Supplementary material for: Unveiling the Fatigue Behavior of 2D Hybrid Organic–Inorganic Perovskites: Insights for Long‐Term Durability
Source: Adv Sci (Weinh). 2023 Jul 6;10(26):2303133. doi: 10.1002/advs.202303133 (PMC10502673; doi:10.1002/advs.202303133)
Supplement: Supplementary file 1 — Supporting Information [file ADVS-10-2303133-s001.pdf]

ADVANCED SCIENCE
Open Access

Supporting Information

for *Adv. Sci.*, DOI 10.1002/advs.202303133

Unveiling the Fatigue Behavior of 2D Hybrid Organic–Inorganic Perovskites: Insights for Long-Term Durability

*Doyun Kim, Eugenia S. Vasileiadou, Ioannis Spanopoulos, Xuguang Wang, Jinhui Yan, Mercouri G. Kanatzidis and Qing Tu**

Unveiling the Fatigue Behavior of 2D Hybrid Organic-Inorganic Perovskites: Insights for Long-Term Durability - Supporting Information

Doyun Kim,[1] Eugenia S Vasileiadou,[2] Ioannis Spanopoulos,[3] Xuguang Wang,[4] Jinhui Yan,[4] Mercouri G. Kanatzidis,[2] Qing Tu[1*]

1. Department of Materials Science & Engineering, Texas A&M University, College Station, TX 77840, USA

2. Department of Chemistry, Northwestern University, Evanston, IL 60201, USA

3. Department of Chemistry, University of South Florida, Tampa, FL 33620, USA

4. Department of Civil & Environmental Engineering, University of Illinois Urbana-Champaign, Urbana, IL 61801-2352

** Corresponding author: Dr. Qing Tu (qing.tu@tamu.edu)*

Section I – Synthesis and Schematics of $(C_4H_9\text{-}NH_3)_2(CH_3NH_3)_2Pb_3I_{10}$ Crystal Structure

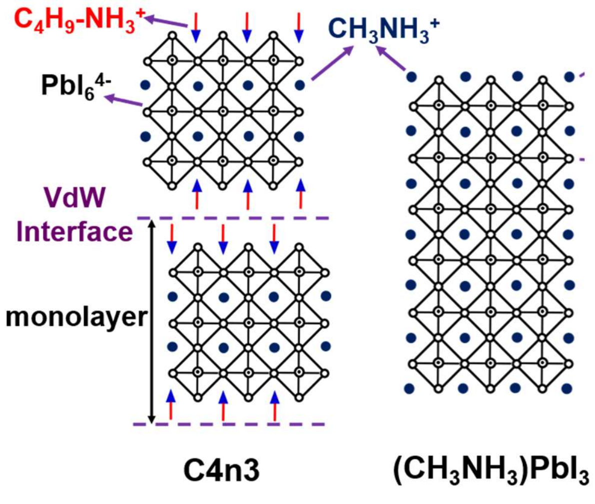

Figure S1. Schematics showing the structures of 2D hybrid organic-inorganic perovskite (HOIP) $(C_4H_9\text{-}NH_3)_2(CH_3NH_3)_2Pb_3I_{10}$ (abbreviated as C4n3) and the structure of the 3D parent HOIP $(CH_3NH_3)PbI_3$.

***I.1. Synthesis*.**

All starting materials for synthesis were purchased commercially and were used without further purification, except for methylamine hydrochloride, which was dried in an oven at 70 ^{0}C overnight. Butylamine 99.5%, Lead(II) oxide powder, <10 μm, ReagentPlus®, ≥99.9% trace metals basis, Methylamine hydrochloride ≥98%, Hypophosphorous acid solution 50 wt. % in H_2O and Hydriodic acid 57 wt. % in H_2O, distilled, stabilized, 99.95%, were purchased from Aldrich. In all cases the published synthetic procedures[1] were modified in order to acquire bigger and thicker single crystals suitable for each type of measurement.

PbO powder (2232 mg, 10 mmol) was dissolved in 57% w/w aqueous HI solution (16 mL) in a 50 mL glass volumetric flask by heating to boiling under constant magnetic stirring for about 5 min, which formed a bright yellow solution. Subsequent addition of solid methylamine hydrochloride (450 mg, 6.67 mmol) to the hot yellow solution initially caused the precipitation of a black powder, which rapidly dissolved under stirring to afford a clear, bright yellow solution. Then 345 μL (3.49 mmol) of butylamine were added to 50% aqueous H_3PO_2 (1.7 mL) and this solution was added to the reaction slowly. The stirring was then discontinued, and the solution was left to cool to room temperature, when brown plate crystals started to crystallize. The precipitation was deemed to be complete after∼4 h. The crystals were isolated by suction filtration and thoroughly dried under reduced pressure. Yield: 1910 mg, (27.2% based on Pb).

I.2. X-ray Diffraction Measurements

Powder X-ray diffraction patterns were collected on a Rigaku Miniflex system (CuKα radiation) operated at 40 kV and 15 mA. A typical scan rate was 15 sec/step with a step size of 0.02 deg.

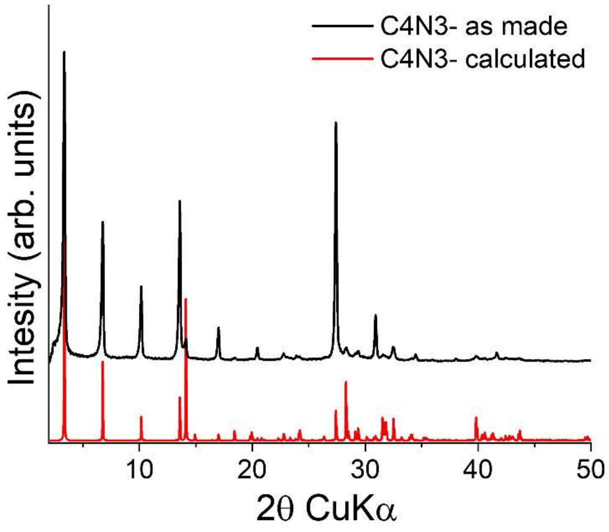

Figure S2. Comparison of the calculated powder X-ray diffraction pattern from the solved single crystal structure of the C4N3 material and experimentally determined one from the as-made crystals.

Section II. Supplementary Methods and Discussions

II.1. AFM Fatigue Control

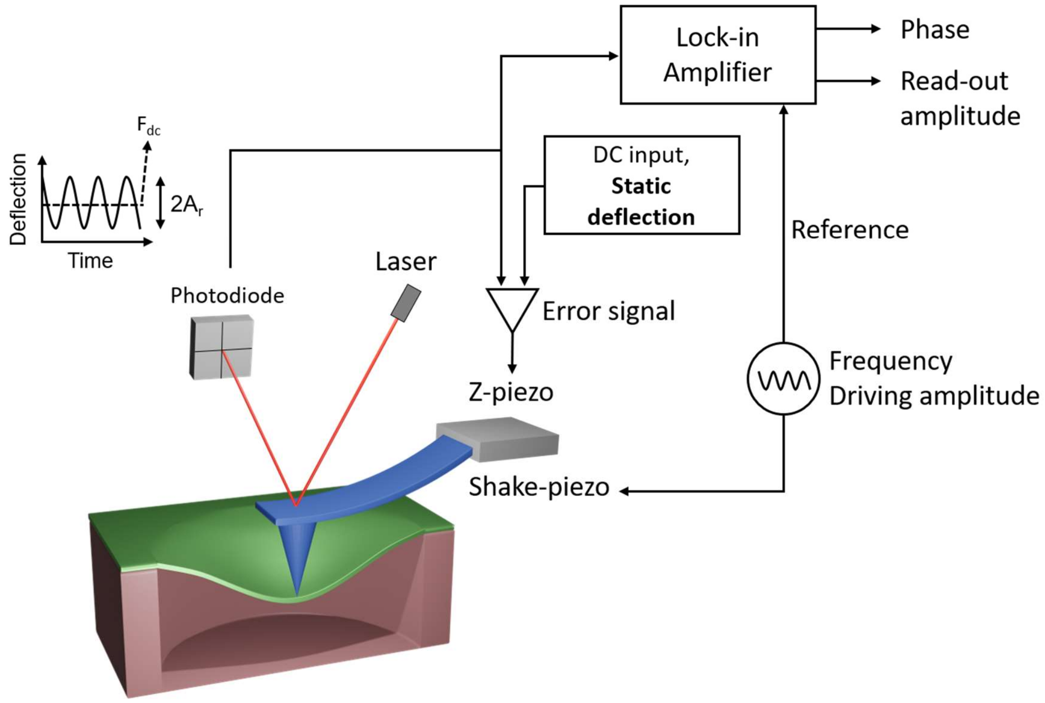

Figure S3. Schematic of the AFM feedback control for fatigue test. For static dwelling test, the tip oscillation amplitude is set to zero (no active driving of the tip oscillation).

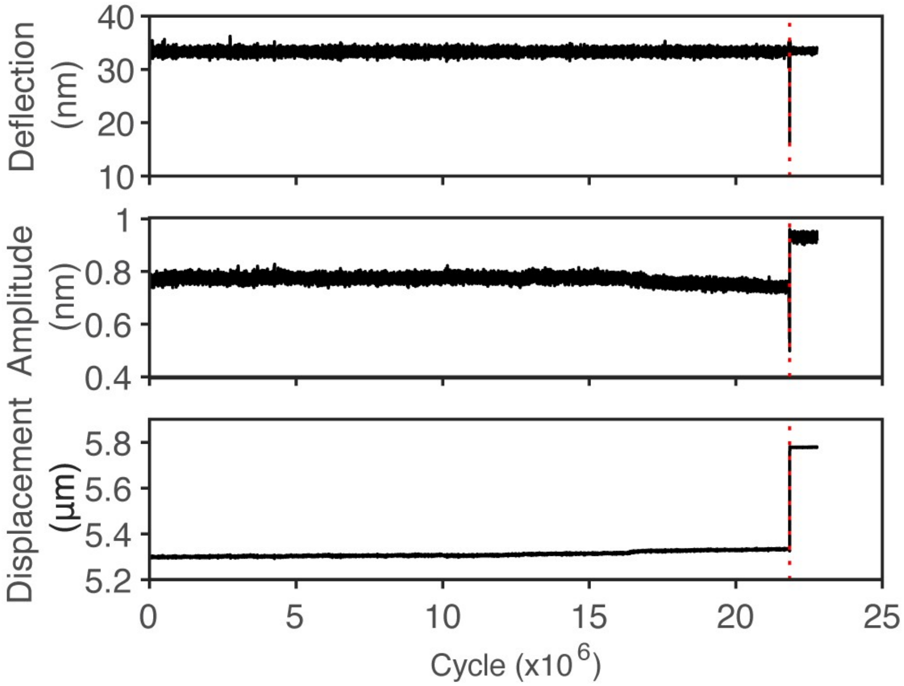

Figure S4. Representative fatigue data showing the signature of fatigue failure during the test (marked by the red dashed line) in cantilever static deflection (top), amplitude (middle) and z-piezo displacement (bottom) channels.

II.2. Resonant Frequency of the Membrane

The first fundamental resonance frequency of a circular, clamped film can be calculated by[2-3]

$$f_1 = \frac{2.405\sqrt{\sigma^{2D}/\rho^{2D}}}{2\pi a} \tag{1}$$

where σ^{2D} is the film tension (in the unit of N/m), ρ^{2D} is area density (in the unit of kg/m^2) and a is the film radius (in the unit of m). σ_0^{2D} can be extracted from the quasi-static force curves (see ***Section II.4*** below) and is typically in the range of 0.01 ~ 0.1 N/m for C4n3 membranes. Taking a~500 nm and ρ^{2D} about 2.67 × 10^{-5} ~ 4.45 × 10^{-5} kg/m^2 for 3 to 5 layers thick membranes,[1, 4-5] f_1 falls in 11.5 ~ 23.4 MHz. This estimation matches very well to the resonant frequency of C4n3 membranes measured by laser interference (Figure S5). With a loading of F_{dc}, the in-plane mean stress σ_{DC}^{2D} will be much larger than σ_0^{2D},[4-6] and hence f_1 will further increase. The frequency of the cyclic force F_{ac} used here (100 kHz) will be away from the resonance of the AFM cantilever and orders of magnitude below the resonance frequency

of the membrane, and thus guarantees the AFM tip always in contact with the membrane during the cyclic loading.[3]

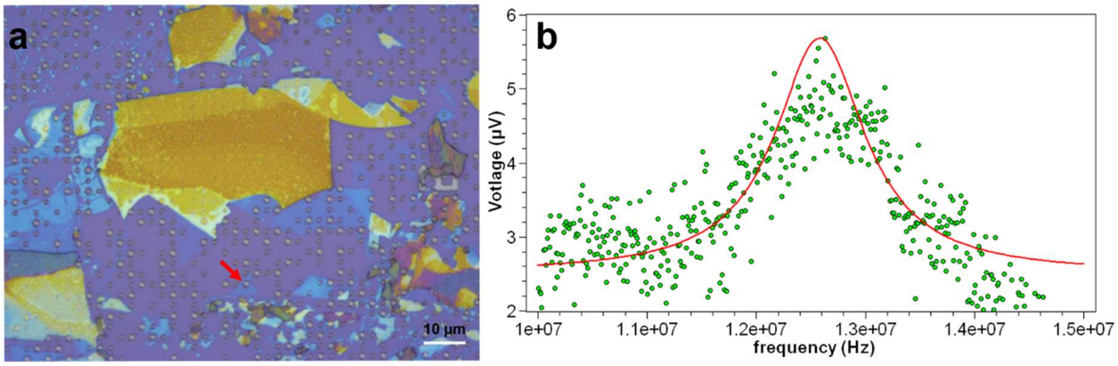

Figure S5. Resonance frequency measurement of a suspended 4-layer C4n3 membrane by laser interference. (a) optical microscopy image of the sample. The tested suspended membrane is marked by the red area. **(b)** Lorentz Fit to the data with actuation voltage 8Vp-p yields $\frac{\omega_0}{2\pi} \sim 12.58$ MHz and FWHM $\frac{\Delta\omega}{2\pi} \sim 1$MHz. The test is conducted with a customized system in Prof. Espinosa Horacio's lab at Northwestern University. Briefly, the silicon sample with the suspended membrane is mounted on and actuated by a PZT disk in vacuum, and a customized laser interference is used to track the amplitude at different driving frequency. Details of the method can be found elsewhere.[7]

II.3. Stress Estimation

We employed finite element method (FEM) simulation similar to estimate the in-plane stresses under cyclic loading conditions using typical experimental conditions (membrane diameter: 1 μm; membrane thickness: 10.4 nm (4 layer C4n3); tip radius: 22.4 nm; $\bar{F}_{\text{fracture}} =$ 57.4 nN; Young's modulus: 7.94 GPa; cyclic force amplitude: 1.5 nN (0.75 nm)). Such method has been widely used to evaluate the stresses in AFM stretching of suspended 2D materials under both static and cyclic loadings.[3, 8] Briefly, the numerical simulations were carried out using the general-purpose FEM software ABAQUS. The membrane was modeled with 3-node triangular shell (S3R) elements. Simulations under different loading conditions were conducted. Fixed boundary conditions were imposed on all nodes on the edge. Based on the mesh sensitivity analysis, the model was meshed with element size of 10 nm. Owing to the axis-symmetry of our simulation setup here, Figure S6 (a) presents a representative in-plane mean stress distribution along the radial direction of the membrane, while the overall stress distribution on the bottom of the membrane is illustrated in Figure S6 (b). Table S1 summarizes the estimated maximum in-plane mean stress (strain) and the stress (strain) amplitude for our test on 4-layer C4n3 membranes at various $F_{\text{dc}}/\bar{F}_{\text{fracture}}$ but fixed tip oscillation amplitude. Although the cyclic stress amplitude varies slightly as we change the applied F_{dc}, the orders of

magnitude change in fatigue lifetime (Figure 2a in the main text) highlights the mean stress effect on the fatigue behavior of 2D HOIPs. Besides, for the same tip oscillation amplitude, higher F_{dc} leads to lower stress amplitude, similar to what is found in graphene fatigue study.[3] The decreasing cyclic stress amplitude should result in an increasing fatigue lifetime at high F_{dc} levels, which contradicts with what we found in our experiments (Figure 2a in the main text). Hence, our results suggest that the effect of this small variation in the cyclic component on the fatigue lifetime should be small and the trend found in Figure 2a in the main text is mainly due to the mean stress effect.

We further compared the applied stress/strain level to those the materials will experience in actual device applications. The reported thermal expansion coefficient for HOIPs is around 5×10^{-5} to 1×10^{-4} /K.[9-11] Thus, the applied strain amplitude (~ 0.1%) in this study corresponds to about 10 to 20 K temperature fluctuation amplitude, which will be commonly found in HOIP-based device applications due to current-induced heating or service environment change.[12-13] For typical HOIP-based flexible electronic device applications, the flexible substrate thickness (h) ranges from 1 μm to tens of μm and the bent radius (R) varies from 0.4 to a few mm.[14-16] The strain levels ($\sim h/2R$)[17] that 2D HOIPs will experience in these flexible electronics applications are thus estimated around 0.02% to 5%. For typical flexible electronic applications, the strain amplitude is engineered through the device architecture design to the lower end of this range (≪ 1%).

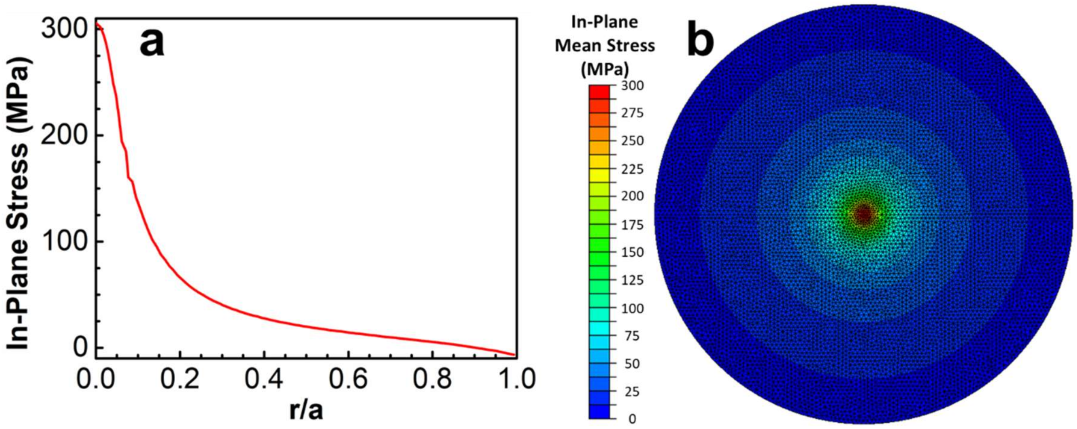

Figure S6. **(a)** Representative radial distribution of the in-plane mean stress estimated by FEM (at $\frac{F_{dc}}{\bar{F}_{fracture}} = 60\%$). Here, the x-axis indicates the distance (**r**) to the center of the membrane normalized by the radius of the suspended membrane (**a** = 500 nm); **(b)** In-plan stress distribution at the bottom face of the specimen.

Table S1. Summary of the maximum in-plane stress, strain and their amplitudes in cyclic loading estimated by FEM.

$F_{dc}/\overline{F}_{\text{fracture}}$	Mean Stress (MPa)	Stress Amplitude (MPa)	Mean Strain (%)	Strain Amplitude (%)
80%	376.8	8.8	3.65	0.086
70%	342.2	9.3	3.32	0.090
60%	305.9	9.7	2.97	0.094
50%	267.6	10.3	2.59	0.10
40%	226.8	11.1	2.20	0.11

II.4. Extraction of the Elastic Modulus and Fracture Strength

Following the method we have established in our lab,[4-6] we could quasi-statically stretch the suspended membrane at the center using the AFM tip and record the force (F) and the displacement of the piezo in the vertical direction Z_{piezo}. The elastic modulus can be extracted from the recorded F *vs.* Z_{piezo} curves[4-6] as summarized briefly below. First, the actual displacement of the membrane δ can be derived by:

$$\delta = Z_{piezo} - \delta_{tip}, \quad (2)$$

where δ_{tip} is the deflection of the AFM tip and is equal to F/k_c (here k_c is the spring constant of the AFM cantilever).

The shape of the curve exhibits a linear regime at low forces and a nonlinear behavior at higher loading forces, regardless of the deformation directions. This can be modeled by an isotropic continuum thin film clamped at the perimeters and loaded at the center.[4, 8, 18-19]The total F *vs.* δ relationship of 2D RP HOIP membranes can be described as[6, 18-20]

$$F - F_0 = \left[\frac{4\pi E_{\parallel} t^3}{3(1-\upsilon^2)a^2}\right](\delta - \delta_0) + \sigma_0^{2D}\pi(\delta - \delta_0) + \frac{q^3 E_{\parallel} t}{a^2}(\delta - \delta_0)^3. \quad (3)$$

Here, $E_{\parallel}$ and σ_0^{2D} are Young's modulus and pretension; υ is the Poisson ratio (υ = 0.23);[6, 21] t and a are the membrane thickness and radius, respectively; (F_0, δ_0) represents the point when the AFM tip just touches the membrane; and $q = 1/(1.05 - 0.15\upsilon - 0.16\upsilon^2)$ is a dimensionless parameter. The first term in Equation (3) represents the contribution from the

bending rigidity of the circular plate.[22-23] The second term arises from the mechanical behavior of a stretched membrane and finally the third term is due to the in-plane stretching of the membrane in reaction to the loading at the center.[8, 19, 22] Hence, at relatively small loading forces, the first two terms dominate because the stiffness of the membrane is mostly caused by the bending rigidity and the pretension (owing to the vdW interaction between the membrane and the SiO_2/Si substrate[4, 8, 24]). The bending rigidity of 2D materials is usually considered as very low[25] and neglected in these mechanical analyses, particularly for atomic or molecularly thin flakes.[8, 24, 26] However, it should be included for thicker flakes like those studied here because the bending rigidity term scales with t^3.[18] At relatively large loading forces, the in-plane stretching begins to prevail, resulting in the cubic shape of the curve. We take $E_{\parallel}$, σ_0^{2D} and (F_0, δ_0) as free parameters and fit the obtained force curves to Equation (3) to extract $E_{\parallel}$ of the material. The great agreement between the fitted curves and the experimental data over the entire force range (Figure S7a) demonstrates the appropriateness of the model.

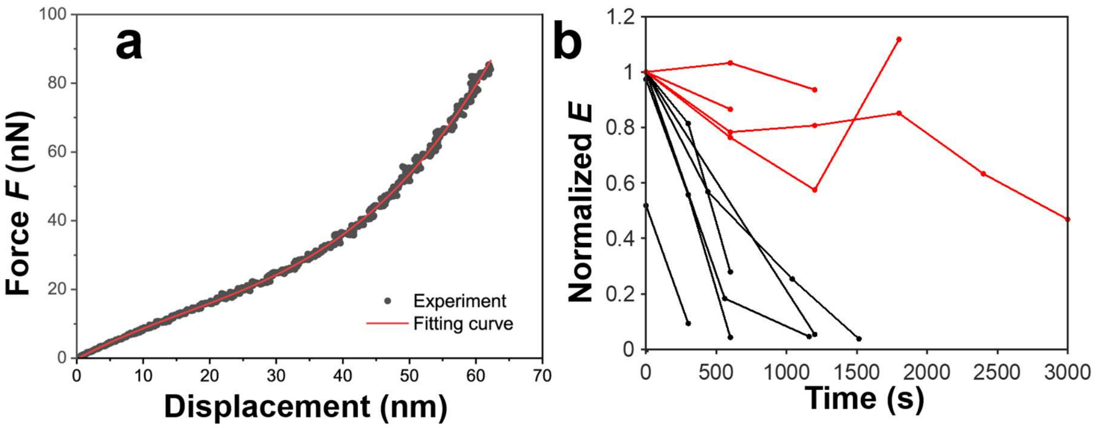

Figure S7. (a) Representative quasi-static stretching force curves fitted by Equation (3); **(b)** Elastic moduli (normalized to the initial elastic modulus) of the tested membranes during fatigue (black) and static dwelling (red) loading.

II.5. Additional Characterizations

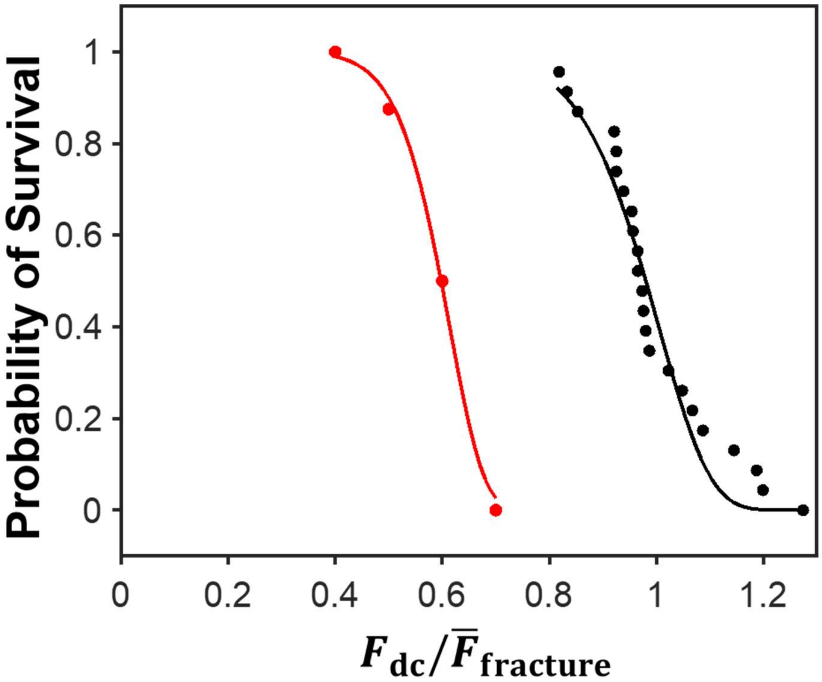

Figure S8. Probability 4-layer C4n3 membranes to survive different forces at quasi-static loading (black) or survive 100 million cycles under cyclic loading with 0.75 nm tip oscillation amplitude (red). The experimental data is fitted by the Weibull distribution (Equation (1) in the main manuscript).

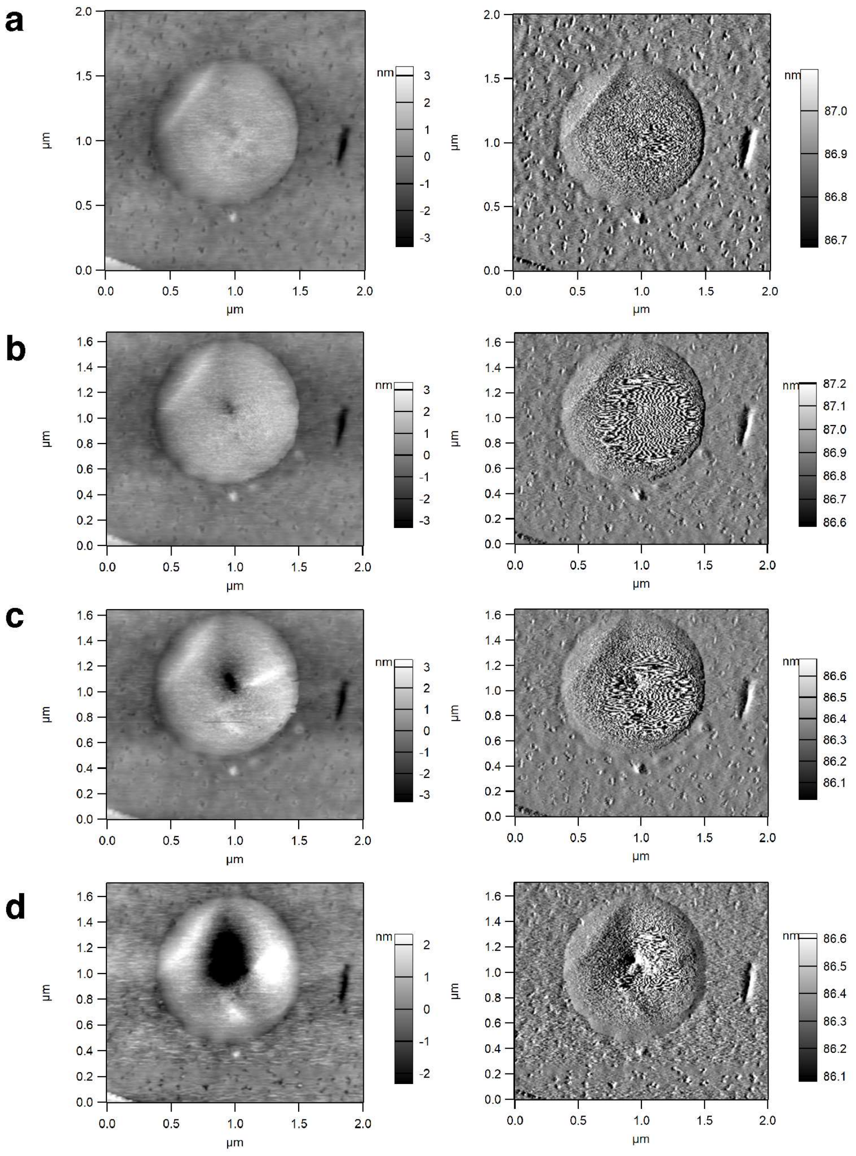

Figure S9. Tapping mode AFM images showing progressive damage in the center of the C4n3 membrane during fatigue test. (a) Before fatigue test, **(b)** after 120 million cycles, **(c)** after 180 million cycles, and **(d)** after 240.006 million cycles (structurally failed). For each row, the left and right images are AFM topography and tapping mode amplitude, respectively. Here, $\frac{F_{dc}}{\bar{F}_{fracture}} = 50\%$ and tip oscillation amplitude = 0.75 nm.

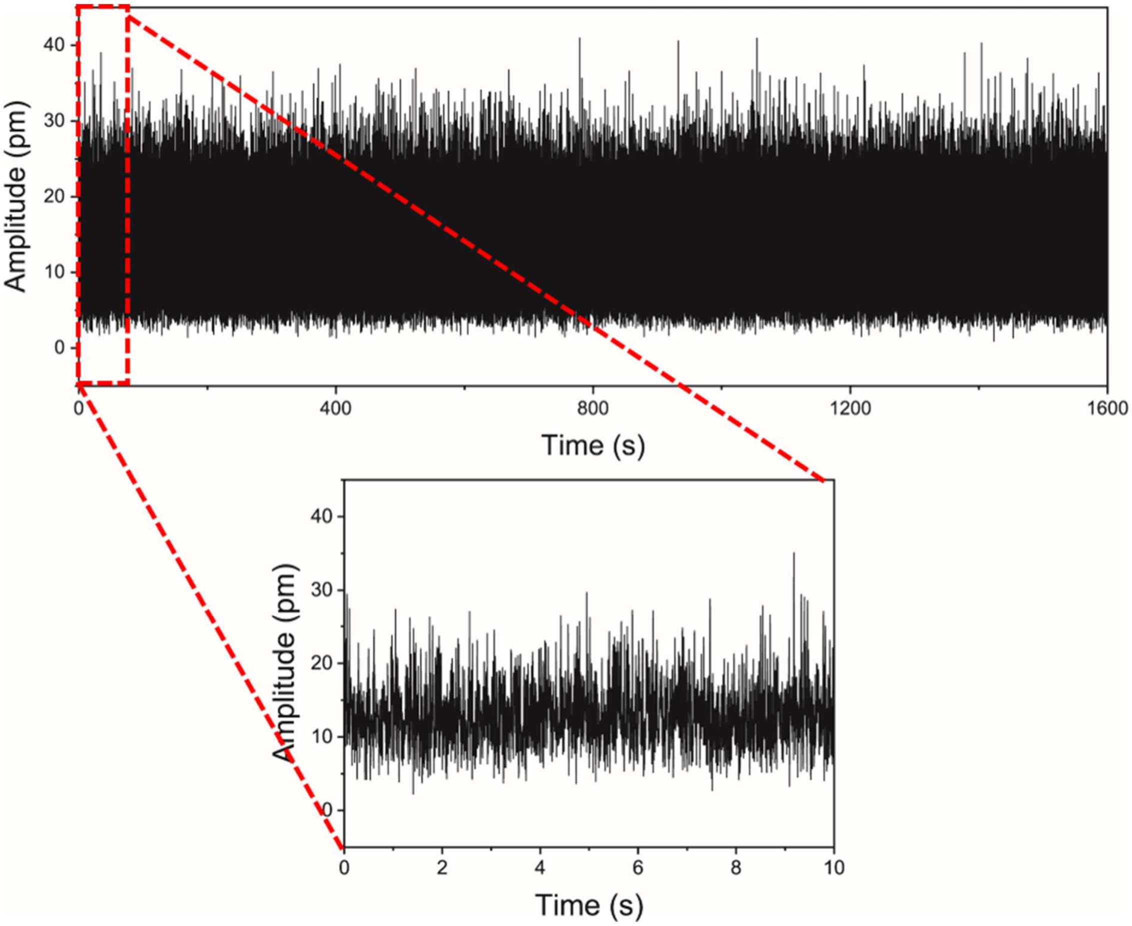

Figure S10. Typical AFM tip oscillation amplitude during static dwelling on a 4-layer C4n3 suspended membrane.

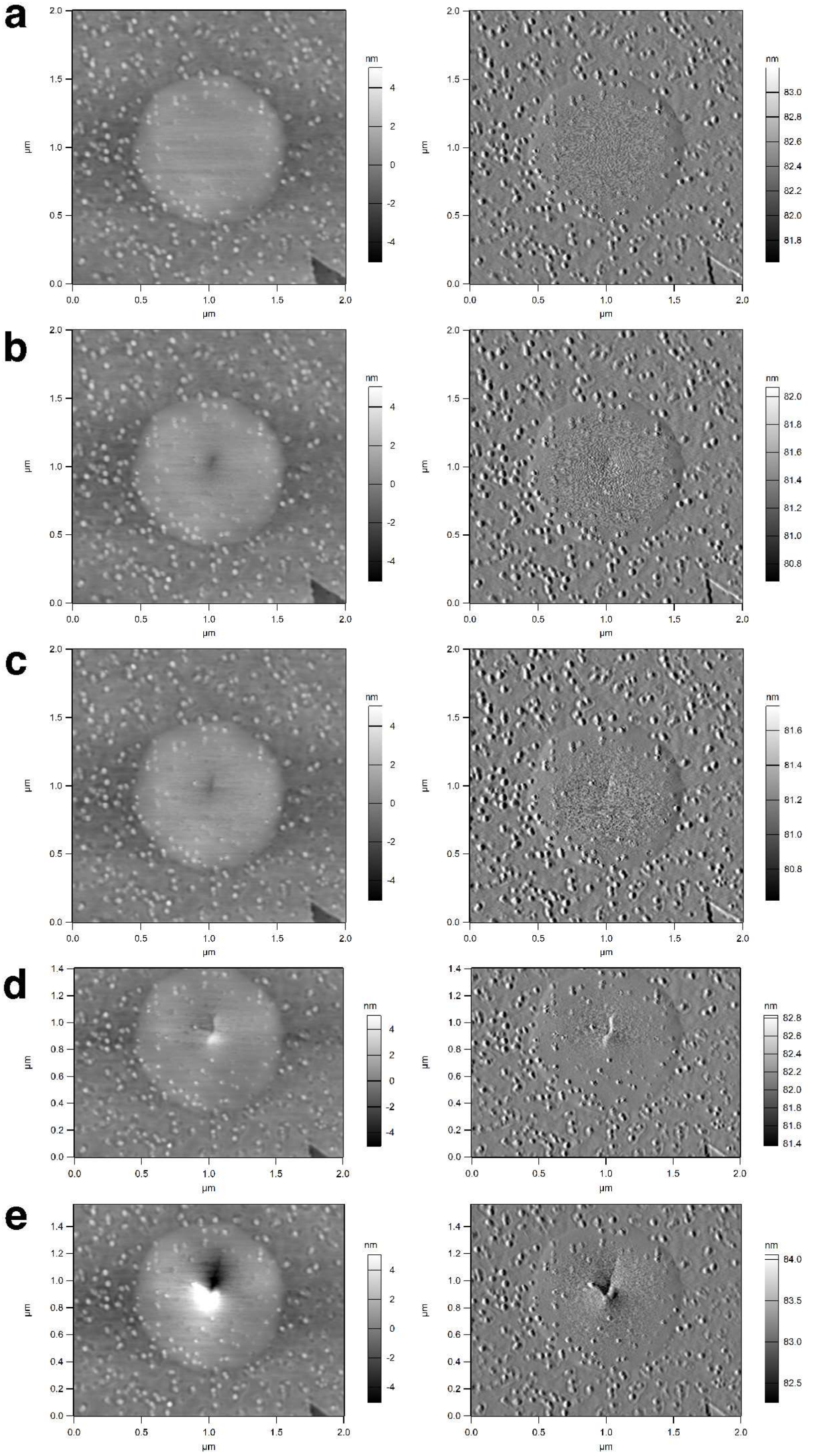

Figure S11. Tapping mode AFM images showing progressive damage in the center of the C4n3 membrane during static dwelling. **(a)** Before static dwelling, **(b)** after 10 mins dwelling, **(c)** after 30 mins dwelling, **(d)** after 60 mins dwelling, and **(e)** after structurally failed. For each row, the left and right images are AFM topography and tapping mode amplitude, respectively. Here $\frac{F_{dc}}{\bar{F}_{fracture}} = 50\%$.

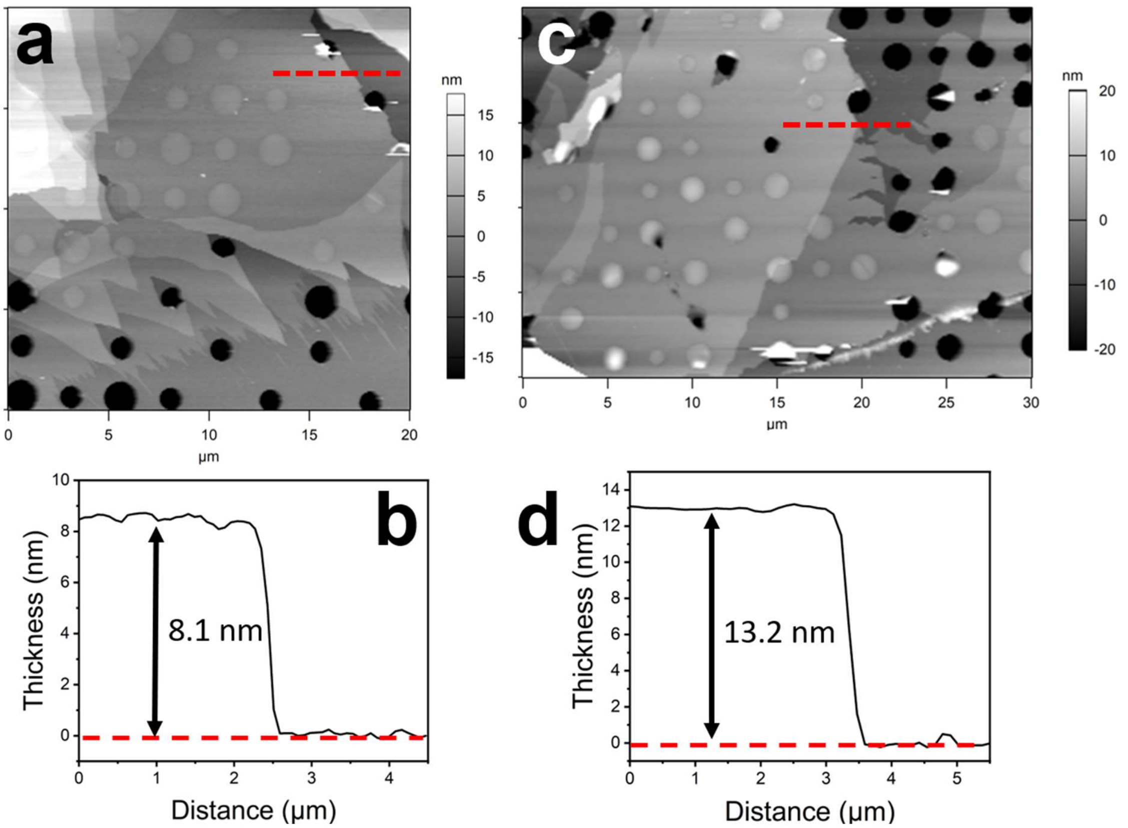

Figure S12. (a) AFM images of representative 3-layer C4n3 flakes**. (b)** Height profile along the red dashed line in **(a)**. **(c)** AFM images of representative 5-layer C4n3 flakes**. (d)** Height profile along the red dashed line in **(c)**.

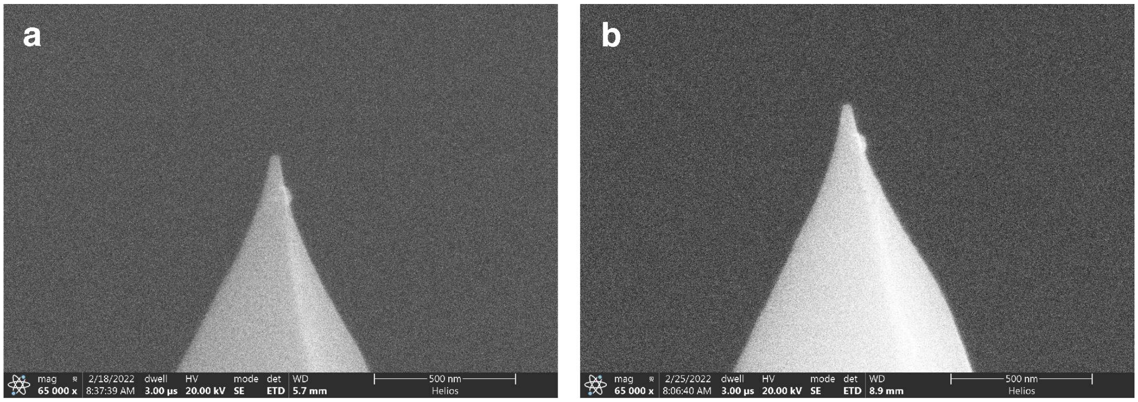

Figure S13. Representative SEM images of the AFM tip radius before (**a**) and after (**b**) the fatigue experiment showing negligible wear. The tip radius remains around 20 nm. Here $\frac{F_{dc}}{\bar{F}_{fracture}} = 40\%$, tip oscillation amplitude = 0.75 nm and the membrane survived 1.52 billion cycles.

References

1. Stoumpos, C. C.; Cao, D. H.; Clark, D. J.; Young, J.; Rondinelli, J. M.; Jang, J. I.; Hupp, J. T.; Kanatzidis, M. G. Ruddlesden–Popper Hybrid Lead Iodide Perovskite 2D Homologous Semiconductors. *Chem. Mater.* **2016,** *28* (8), 2852-2867.
2. Meirovitch, L. *Principles and Techniques of Vibrations*. Prentice-Hall International, Inc: **1997**.
3. Cui, T.; Mukherjee, S.; Sudeep, P. M.; Colas, G.; Najafi, F.; Tam, J.; Ajayan, P. M.; Singh, C. V.; Sun, Y.; Filleter, T. Fatigue of Graphene. *Nat. Mater.* **2020,** *19* (4), 405-411.
4. Tu, Q.; Spanopoulos, I.; Yasaei, P.; Stoumpos, C. C.; Kanatzidis, M. G.; Shekhawat, G. S.; Dravid, V. P. Stretching and Breaking of Ultrathin 2D Hybrid Organic–Inorganic Perovskites. *ACS Nano* **2018,** *12* (10), 10347-10354.
5. Kim, D.; Vasileiadou, E. S.; Spanopoulos, I.; Kanatzidis, M. G.; Tu, Q. Abnormal In-Plane Thermomechanical Behavior of Two-Dimensional Hybrid Organic–Inorganic Perovskites. *ACS Appl. Mater. Interfaces* **2023,** *15* (6), 7919-7927.
6. Kim, D.; Vasileiadou, E. S.; Spanopoulos, I.; Kanatzidis, M. G.; Tu, Q. In-Plane Mechanical Properties of Two-Dimensional Hybrid Organic–Inorganic Perovskite Nanosheets: Structure–Property Relationships. *ACS Appl. Mater. Interfaces* **2021,** *13* (27), 31642-31649.
7. Nathamgari, S. S. P.; Dong, S.; Medina, L.; Moldovan, N.; Rosenmann, D.; Divan, R.; Lopez, D.; Lauhon, L. J.; Espinosa, H. D. Nonlinear Mode Coupling and One-to-One Internal Resonances in a Monolayer WS_2 Nanoresonator. *Nano Lett.* **2019,** *19* (6), 4052-4059.
8. Lee, C.; Wei, X.; Kysar Jeffrey, W.; Hone, J. Measurement of the Elastic Properties and Intrinsic Strength of Monolayer Graphene. *Science* **2008,** *321* (5887), 385-388.
9. Ramirez, C.; Yadavalli, S. K.; Garces, H. F.; Zhou, Y.; Padture, N. P. Thermo-Mechanical Behavior of Organic-Inorganic Halide Perovskites for Solar Cells. *Scr. Mater.* **2018,** *150*, 36-41.
10. Haeger, T.; Heiderhoff, R.; Riedl, T. Thermal Properties of Metal-Halide Perovskites. *J. Mater. Chem. C* **2020,** *8* (41), 14289-14311.
11. Seo, J.; McGillicuddy, R. D.; Slavney, A. H.; Zhang, S.; Ukani, R.; Yakovenko, A. A.; Zheng, S.-L.; Mason, J. A. Colossal Barocaloric Effects with Ultralow Hysteresis in Two-Dimensional Metal–Halide Perovskites. *Nat. Commun.* **2022,** *13* (1), 2536.
12. Boyd, C. C.; Cheacharoen, R.; Leijtens, T.; McGehee, M. D. Understanding Degradation Mechanisms and Improving Stability of Perovskite Photovoltaics. *Chem. Rev.* **2019,** *119* (5), 3418-3451.
13. Holzhey, P.; Saliba, M. A Full Overview of International Standards Assessing the Long-Term Stability of Perovskite Solar Cells. *J. Mater. Chem. A* **2018,** *6* (44), 21794-21808.
14. Ma, Y.; Lu, Z.; Su, X.; Zou, G.; Zhao, Q. Recent Progress Toward Commercialization of Flexible Perovskite Solar Cells: From Materials and Structures to Mechanical Stabilities. *Adv. Energy Sustain. Res.* **2023,** *4* (1), 2200133.
15. Zhang, J.; Zhang, W.; Cheng, H.-M.; Silva, S. R. P. Critical Review of Recent Progress of Flexible Perovskite Solar Cells. *Mater. Today* **2020,** *39*, 66-88.
16. Tang, G.; Yan, F. Recent Progress of Flexible Perovskite Solar Cells. *Nano Today* **2021,** *39*, 101155.
17. Tu, Q.; Spanopoulos, I.; Hao, S.; Wolverton, C.; Kanatzidis, M. G.; Shekhawat, G. S.; Dravid, V. P. Probing Strain-Induced Band Gap Modulation in 2D Hybrid Organic–Inorganic Perovskites. *ACS Energy Lett.* **2019,** *4* (3), 796-802.
18. Andres, C. G.; Menno, P.; A., S. G.; J., v. d. Z. H. S.; Nicolás, A.; Gabino, R. B. Elastic Properties of Freely Suspended MoS_2 Nanosheets. *Adv. Mater.* **2012,** *24*, 772-775.
19. Komaragiri , U.; Begley, M. R.; Simmonds, J. G. The Mechanical Response of Freestanding Circular Elastic Films Under Point and Pressure Loads. *J. Appl. Mech.* **2005,** *72* (2), 203-212.

20. Harbola, V.; Xu, R.; Crossley, S.; Singh, P.; Hwang, H. Y. Fracture and Fatigue of Thin Crystalline $SrTiO_3$ Membranes. *Appl. Phys. Lett.* **2021,** *119* (5), 053102.
21. Gao, H.; Wei, W.; Li, L.; Tan, Y.; Tang, Y. Mechanical Properties of a 2D Lead-Halide Perovskite, $(C_6H_5CH_2NH_3)_2PbCl_4$, by Nanoindentation and First-Principles Calculations. *J. Phys. Chem. C* **2020,** *124* (35), 19204-19211.
22. Timoshenko, S.; Woinowsky-Krieger, S. *Theory of Plates and Shells*. McGraw-Hill: New York, **1959**.
23. Landau, L. D. a. E. M. L. *Theory of Elasticity / Translated from the Russian by J. B. Sykes and W. H. Reid*. Pergamon: London, **1959**; p 1-177.
24. Bertolazzi, S.; Brivio, J.; Kis, A. Stretching and Breaking of Ultrathin MoS_2. *ACS Nano* **2011,** *5*, 9703-9709.
25. Han, E.; Yu, J.; Annevelink, E.; Son, J.; Kang, D. A.; Watanabe, K.; Taniguchi, T.; Ertekin, E.; Huang, P. Y.; van der Zande, A. M. Ultrasoft Slip-Mediated Bending in Few-Layer Graphene. *Nat. Mater.* **2020,** *19* (3), 305-309.
26. Falin, A.; Cai, Q.; Santos, E. J. G.; Scullion, D.; Qian, D.; Zhang, R.; Yang, Z.; Huang, S.; Watanabe, K.; Taniguchi, T.; Barnett, M. R.; Chen, Y.; Ruoff, R. S.; Li, L. H. Mechanical Properties of Atomically Thin Boron Nitride and the Role of Interlayer Interactions. *Nat. Commun.* **2017,** *8* (1), 15815.
